# Microscopic Pillars and Tubes Fabricated by Using Fish Dentine as a Molding Template

**DOI:** 10.3390/ijms150914909

**Published:** 2014-08-25

**Authors:** Weiqun Li, Xiaowei Liu, Yang Lu, Haimin Yao

**Affiliations:** 1 The Hong Kong Polytechnic University Shenzhen Research Institute, Shenzhen 518057, China; E-Mail: david.w.li@connect.polyu.hk; 2 Department of Mechanical Engineering, The Hong Kong Polytechnic University, Hung Hom, Kowloon, Hong Kong, China; 3 Department of Mechanical and Biomedical Engineering, City University of Hong Kong, Kowloon Tong, Hong Kong, China; E-Mails: xwliu5-c@my.cityu.edu.hk (X.L.); yanglu@cityu.edu.hk (Y.L.); 4 Centre for Advanced Structural Materials (CASM), City University of Hong Kong, Hong Kong, China

**Keywords:** adhesion, contact mechanics, hierarchical structures, biomimetics

## Abstract

Biomaterials in nature exhibit delicate structures that are greatly beyond the capability of the current manufacturing techniques. Duplicating these structures and applying them in engineering may help enhance the performance of traditional functional materials and structures. Inspired by gecko’s hierarchical micro- and nano-fibrillar structures for adhesion, in this work we fabricated micro-pillars and tubes by adopting the tubular dentine of black carp fish teeth as molding template. The adhesion performances of the fabricated micro-pillars and tubes were characterized and compared. It was found that the pull-off force of a single pillar was about twice of that of the tube with comparable size. Such unexpected discrepancy in adhesion was analyzed based on the contact mechanics theories.

## 1. Introduction

Decades of study on the biological attachment systems showed that micro- and nano-fibrillar structures could enhance adhesion [1]. For instance, it was demonstrated that the excellent climbing capability of gecko was essentially due to the intermolecular adhesion (van der Waals force) between millions of nanoscaled fibrils on its feet and the solid surface it attaches on [2]. The adhesion enhancement due to fibrillar structures works even in aquatic environment. Northern clingfish adheres firmly to surface under water through micro-fibrillar structures. The adhesion force could reach up to 230 times of its body weight [3]. To get a better understanding on the mechanism of adhesion enhancement by fibrillar structure, a “contact splitting” principle [4] was proposed, predicting that adhesion force would increase by a factor of *n*^1/2^ if one larger contact is divided into *n* smaller contacts. This theory may explain why in nature heavier animals have finer fibrillar structures for attachment [5]. In addition to the size, other parameters, such as fibril geometry [6,7], tilt angle [7,8,9,10,11,12], contact shape [6,13,14], humidity [15] and hierarchical structure [16], also play important roles in determining robust adhesion and easy detachment [17]. A design map was established to show the effects of these parameters on the adhesion, giving a practical guideline for the design of manmade micro- and nano-fibrillar structures for adhesion [18].

Inspired by these natural fibrillar structures, people tried out to fabricate micro- and nano-fibrillar structures for potential application as artificial adhesive systems. So far, several fabrication techniques have been applied, such as mold casting [19], nanodrawing [20], chemical-vapor deposition [21] and UV nano-embossing [22]. Among them, mold casting is one of the most widely used approaches in which liquid polymers and curing agents are injected into tubular templates. After curing of the polymers and removal of the templates, polymeric fibrillar structures are obtained. The structure of the obtained fibrils precisely duplicates that of the tubules. Therefore, having a tubular mold of high quality is an essential step for the fabrication of fibrillar structures. Nowadays, the prevailing techniques used to fabricate microscopic tubular structures include three-dimensional (3D) lithographic patterning [19] and anodic oxidation [23,24], both of which involve either subtle conditions of chemical reaction or sophisticated experimental facilities. Finding environmentally-friendly and cost-effective templates is of great interest and value to the community of biomimetic and functional materials. In this work, an attempt was made to do this by resorting to natural materials.

In nature, biomaterials [25] tend to adopt delicate structures at multiple length scales to achieve unique properties and functions. It could be an efficient and practical way to apply biomaterials and structures as templates to duplicate both their structures and the consequent properties and functions. For example, the alumina replicas of the wing of a *Morpho* *peleides* butterfly inherited the excellent optical reflection properties and could be used as waveguide and beam splitter [26]. The nanostructured surfaces duplicated from the compound eyes of household fly showed superior anti-reflection properties, implying significant promise in solar energy harvesting [27]. Artificial leaves mimicking rice and lotus leaves were fabricated by a two-step phase-separation micromolding process, exhibiting anisotropic wettability and the superhydrophobicity, respectively [28]. In order to fabricate micro fibrillar-structures for adhesion, templates with tubular structures are needed. Coincidently, in our recent study on the teeth of black carp fish [29], we found that the dentine of black carp teeth exhibits delicate tubular structures, making it an ideal template for the fabrication of fibrillar structures. In this paper, fibrillar structures for adhesion use were fabricated by adopting dentine of black carp as molding templates. The adhesion properties of the fabricated fibrillar structures were studied and compared with the theoretical estimations.

## 2. Results and Discussion

### 2.1. Tubular Microstructures in Dentine of Black Carp Fish

**Figure 1:**
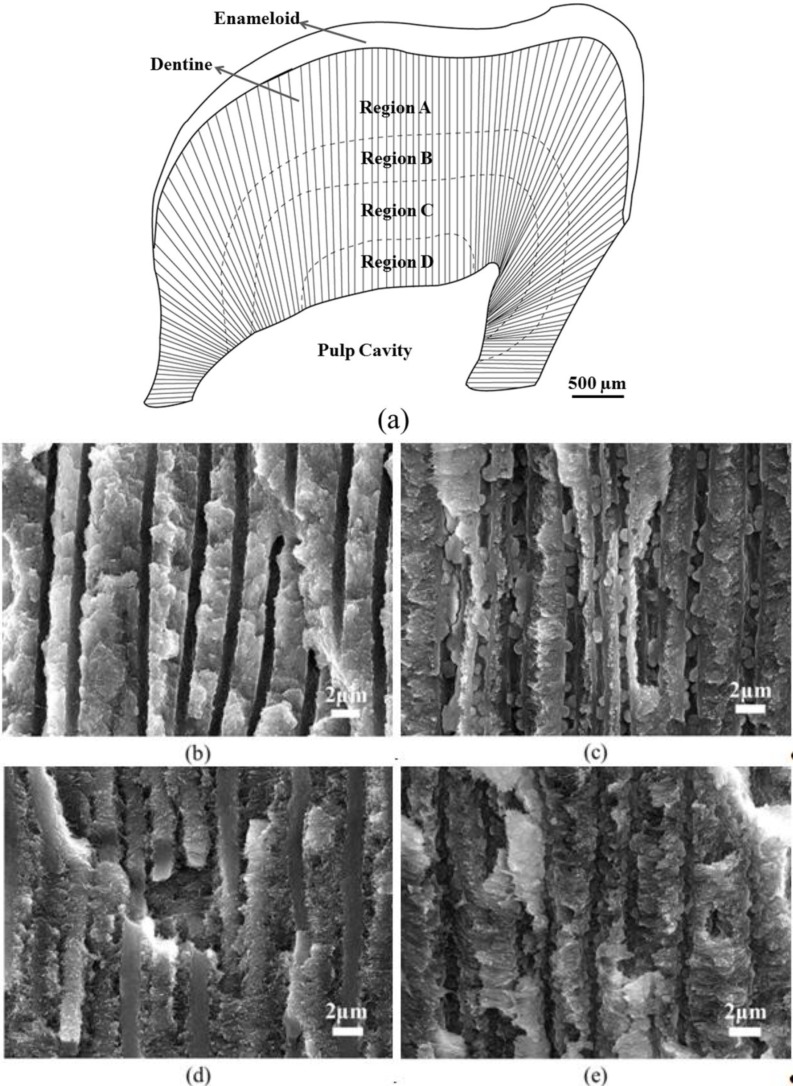
(**a**) Schematic of the longitudinal section of a pharyngeal tooth of black carp and (**b**–**e**) SEM images of a longitudinal section of black carp teeth from regions A–D indicated in (**a**).

As it happens with the teeth of other animals such as mammals, the pharyngeal teeth of black carp (*Mylopharyngodon piceus*), one of the autochthonous fishes inhabiting in East China, exhibit a two-layer structure with an outer enameloid layer and inner dentine layer (Figure 1a). Within the dentine layer, the tubules are aligned along the radial direction from the inner side to the outer side. It was reported that these tubular structures result from the retreat of odontoblasts from the formed dentine towards the pulp when the dentine was made up [30]. Scanning Electron Microscopy (SEM) characterization indicated that the tubular structure of dentine exhibits distinct structural features at different depths. Roughly, it can be divided into four regions (Figure 1a): in Region A, close to the enameloid layer, the tubular structure is completely hollow (Figure 1b). This region covers a thickness of around 700 μm. In Region B with a thickness of about 500 μm, protrusions of 600 nm diameter appear on the walls of the tubules (Figure 1c). Figure 1d shows the subsequent Region C, in which the tubules are not hollow but filled with solid cores or fillers. In the most inner region near the pulp cavity, Region D, the tubular structures appear hollow again (Figure 1e). In addition, the walls of the tubules of Region D seem rougher than those of Region B. It is still unclear why nature adopts such sophisticated tubular structures in the dentine of black carp fish.

**Figure 2:**
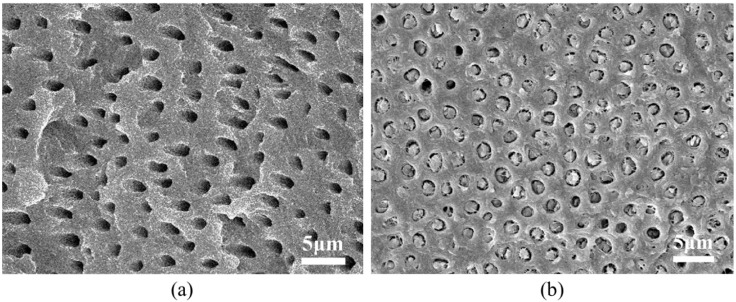
SEM images of transverse sections of dentine in (**a**) Region A; and (**b**) Region C.

To shed more light on the tubular structure, SEM imaging of the transverse sections of Regions A and C was carried out. It can be seen from Figure 2 that the hollow micro-tubules in Region A have a 1–2 μm diameter and an inter-tubule spacing of 1–3 μm. In contrast, in the Region C, the micro-tubules contain solid fillers with diameter ~1.2 μm. The fillers do not completely occupy the space in the tubules. Instead, there is some free space between the fillers and inner wall of the tubules.

**Figure 3:**
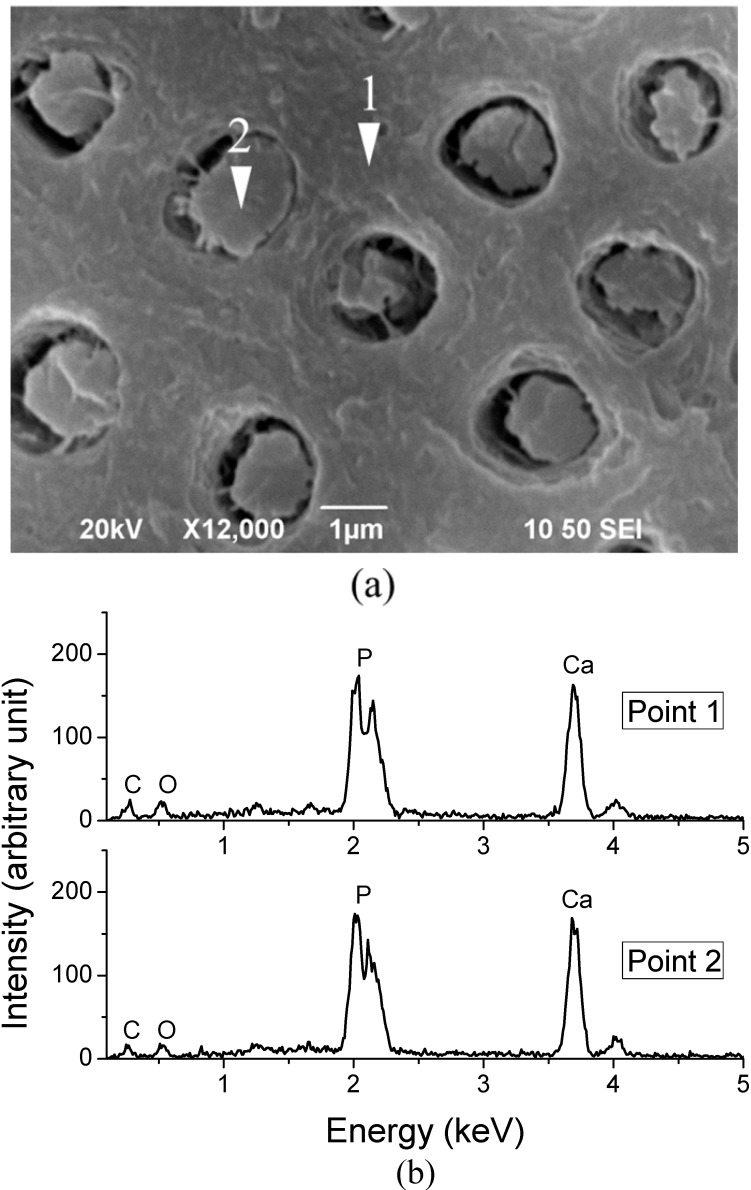
(**a**) Locations from where X-ray spectroscope analysis (EDX)spectra were obtained: Point 1 is on the dentine layer; Point 2 is located on the filler that is encapsulated in the tubules of dentine; (**b**) EDX spectra obtained at Points 1 and 2.

Energy-dispersive X-ray spectroscope analysis (EDX) was conducted to identify the composition of fillers that are encapsulated in the tubules. Figure 3b shows the EDX spectra collected from Points 1 and 2 (Figure 3a). It can be seen that the EDX spectra at Points 1 and 2 have similar patterns, with phosphorus (P), calcium (Ca), carbon (C) and oxygen (O) being the main constituent elements. This result confirms that fillers are composed by the same constituents as those of dentine.

### 2.2. Fabricated Micro-Pillars and Tubes

**Figure 4:**
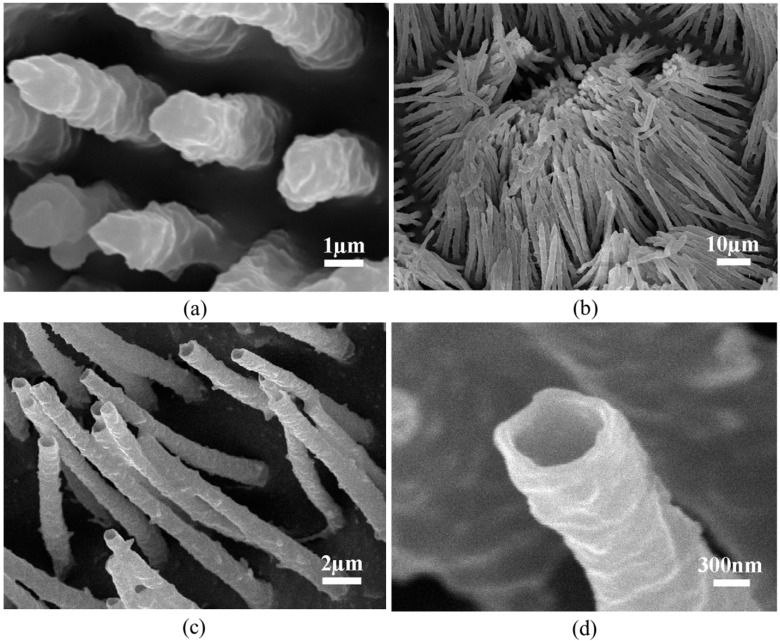
SEM images of the fabricated (**a**,**b**) micro-pillars and (**c**,**d**) micro-tubes.

Figure 4 shows the micro-pillar and tube arrays fabricated using black carp dentine as the template. The pillar diameter is around 1.5 µm and the inter-pillar spacing is 1–3 µm, which are in agreement with the dimensional features of the tubular structures in the dentine template (Figure 4a). As it happened with the fiber arrays fabricated using other techniques [22,31], bunching phenomenon was also observed in our product when longer micro-pillars were fabricated (Figure 4b). In addition to micro-pillars, micro-tubes were also obtained by using templates incised from Region C of the dentine (Figure 4c). The diameter and wall thickness of the tubes are 1–2 µm and 100–300 nm, respectively (Figure 4d).

**Figure 5:**
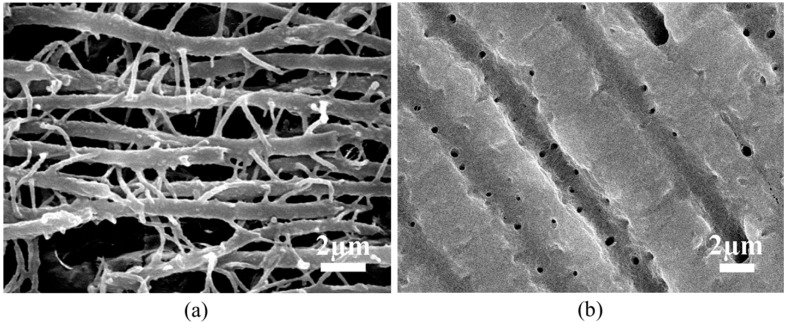
SEM images of the(**a**) fabricated hierarchical fibrillar structure and (**b**) hierarchical tubular structure in black carp dentine.

Surprisingly, a two-leveled hierarchical fibrillar structure was obtained (Figure 5a). This is basically due to the nano pores branching from the micro main tubules, as shown in Figure 5b. The diameter of these nano pores ranges from 100 to 300 nm, justifying the diameter of the branching fibrils shown in Figure 5a.

### 2.3. Adhesion Properties of Single Micro-Pillar and Tube

**Figure 6:**
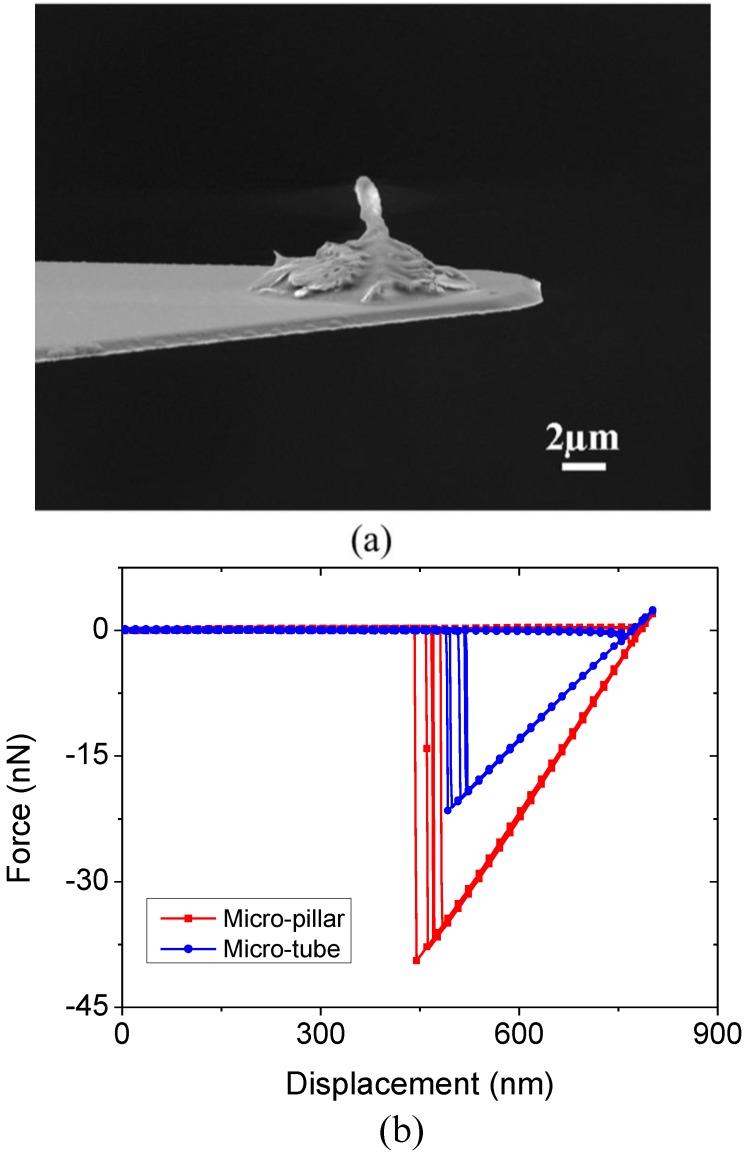
(**a**) SEM image of the micro-pillar attached on the tipless Atomic Force Microscopy (AFM) probe; (**b**) Measured adhesion curves of single fabricated micro-pillar and micro-tube.

The tip shape of fibrillar structures was found to play an important role in determining their adhesion performance [6,7,10,11]. In order to compare the adhesive performances of the fabricated micro-pillar and tube, measurement of adhesion force for a single micro-pillar and tube was carried out. The aspect ratios of the selected micro-pillar and tube are around 1:2.5. The measured force-displacement curves are shown in Figure 6b. It can be seen that the pull-off force for micro-pillar is 37.3 ± 2.2 nN (*n* = 5), while 20.3 ± 1.2 nN (*n* = 5) for micro-tube. By taking the cross section area as the apparent contact area, the strengths (or pull-off stress) are estimated to be around 21.1 and 24.9 kPa for a single micro-pillar and tube respectively, which are comparable to the values of adhesive structures with other tip shapes [6,32].

In contact mechanics, the Johnson-Kendall-Roberts (JKR) theory [33] indicates that the pull-off force between an elastic sphere and a substrate is *F_c_* = 1.5π*R*∆γ, where *R* stands for the radius of the sphere and ∆γ is the adhesion energy. For the micro-pillar, if it is assumed that the pillar tip is a semi-sphere with radius of 750 nm, the work of adhesion is estimated to be:

∆γ = *F_c_*/1.5π*R* = 10.6 mJ/m^2^ (1)

which is consistent with the previous measurements of van der Waals interaction between Polydimethylsiloxane (PDMS) and glass [34].

For the adhesion between tube and substrate, the pull-off force can be theoretically estimated by considering the end of the tube as a donut-shaped ring with outer and inner radii being *R*_1_ and *R*_2_ respectively. Due to the similarity between the axi-symmetric problem and plane strain problem in mechanics, the pull-off force between a micro-tube and a flat substrate then can be equated to the pull-off force of a 2D (plane strain) cylinder of unit length multiplying the circumference of the ring [35]. Therefore, the pull-off force between the tube and substrate is given by the expression.

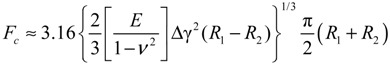
(2)
where *E* and *ν* are the Young’s Modulus and Poisson’s Ratio of PDMS. Taking *E* = 750 kPa [36], *ν =* 0.5, ∆γ = 10.6 mJ/m^2^, *R*_1_ = 750 nm and *R*_2_ = 550 nm, the pull-off force is estimated to be around 159 nN, which is much higher than the experimental value 20 nN. The discrepancy between the theoretical estimation and experimental measurement of the micro-tube may be attributed to the tilt of the tube during adhesion, which results in incomplete contact with the substrate and a much lower pull-off force. Similar reduction of pull-off force due to tilting has been also reported for the fibril with flat tip but not for fibrils with spherical tip [7,12]. Therefore, tubes may not necessarily lead to stronger adhesion in comparison to pillars, unless a complete contact is ensured which, however, is difficult to get in practice.

## 3. Experimental Section

### 3.1. Fabrication of Templates

The teeth of black carp were cut from the pharyngeal bone by water-lubricated low-speed corundum saw (Minitom, Struers). After cleaning with deionized water and drying in air, the samples were embedded in epoxy resin. After curing in air for 10 h, the samples were incised into 300–500 μm thick slices with a diamond saw. The incisions were made in such a way that the sections were basically perpendicular to the longitudinal direction of the tubules in dentine. After ultrasonic cleaning within 10 s, rinsing with deionized water and drying in air, the slices were ready for use as the templates of mold casting.

### 3.2. SEM Characterization and EDX Analysis

All the specimens were sputtered with a gold coating prior to SEM characterization (JSM-6490, JOEL and Quanta 450, FEI) with accelerating voltage equal to 20 kV. For dentine, SEM characterization was made on both longitudinal and transverse sections. In order to identify the chemical components of the filler encapsulated in the tubules of dentine, EDX analysis was conducted.

### 3.3. Fabrication of Micro-Pillars and Tubes Using Mold Casting

**Figure 7:**
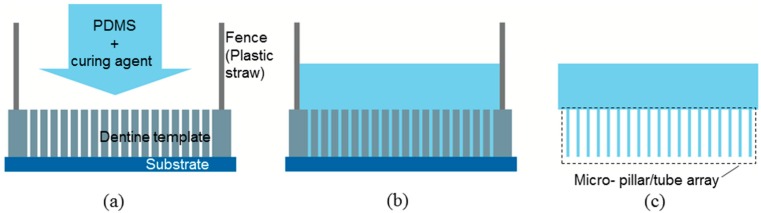
Schematics of fabrication process: (**a**) casting; (**b**) curing at 70°C for 100 min; (**c**) demolding.

PDMS and curing agent (Sylgard 184) were mixed with volume ratio of 10:1 at 25 °C. The mixture was placed in vacuum for 2 min to eliminate the entrapped air bubbles. The dentine template was placed on a substrate. The PDMS mixture was poured into the template with the help of a fence (a short piece of plastic straw), which was placed onto the top of the template to avoid the PDMS from draining away (Figure 7a). The whole assembly was then put into a vacuum oven for curing, at 70 °C, for 100 min (Figure 7b). After cooling down at room temperature, the dentine template was etched away by immersing the assembly into the 37 wt % hydrochloric acid solution for 8 h, giving rise to the micro-pillars or tubes (Figure 7c).

### 3.4. Adhesion Measurement

A micromanipulator (450PM, the Micromanipulator Co., Carson City, NV, USA) was used to pick single micro-pillar or tube from the array, which was then attached to the end of a tipless Atomic Force Microscopy (AFM) probe using silver epoxy (No. 16043, Ted Pella, Inc., Redding, CA, USA) (Figure 6a, for the pillar). The spring constant of the probe was calibrated by thermal tune method after the determination of deflection sensitivity [37]. After mounting the modified probe on the AFM (SPM Multimode 8, Bruker Corporation, Billerica, MA, USA), adhesion force between the micro-pillar/tube and glass slide was measured under contact mode. Each measurement was repeated by 5 times at different positions on the glass slide. Preload of ~2.0 nN was applied in our measurements.

## 4. Conclusions

In summary, in this paper micro-pillars and tubes were fabricated using mold casting technology with dentine of black carp as the template. In comparison to the traditional chemical synthetic approaches [19,20,21,22,24], our dentine templates are easier to prepare and allow us to obtain the micro-pillars and tubes at the same time. Our result showed that the micro-pillar exhibits higher pull-off force in comparison to the tube of similar diameter. This finding deviates from the theoretical estimation based on contact mechanics. Such discrepancy can be attributed to the sensitivity of the pull-off force of microscopic tube to the angle at which it makes contact with the substrate. Therefore, the tip geometry of a single fibril and the statistical distribution of tilting angles in a fibril array matter and deserve more attention in the design of fibrillar structures for adhesion.
